# Magnetic Resonance Imaging Scan of the Brain After Mild COVID-19 Infection

**DOI:** 10.7759/cureus.34229

**Published:** 2023-01-26

**Authors:** Makoto Ohtake, Jun Suenaga, Taisuke Akimoto, Hisataro Ikeuchi, Ayumu Muroya, Hiroyuki Ohata, Yoshihiro Kubota, Masaaki Chiku, Tomoaki Hamano, Tetsuya Yamamoto

**Affiliations:** 1 Department of Neurosurgery, Yokohama City University Hospital, Yokohama, JPN; 2 Department of Smart Brain Screening, Smartscan, Inc., Tokyo, JPN; 3 Department of Neurosurgery, Medical Check Studio, Tokyo, JPN

**Keywords:** covid-19 antibody test, brain screening, mild covid-19 infection, magnetic resonance angiography (mra), magnetic resonance imaging ( mri)

## Abstract

Purpose: There have been several reports of central nervous system impairments associated with severe coronavirus disease 2019 (COVID-19) infection on head magnetic resonance imaging and angiography (MRI/A). However, head MRI/A is rarely performed in mild cases, and there have been few reports on intracranial changes after COVID-19 infection in these cases. Here, we report a comparative examination of the findings seen in common head MRI/A sequences in mild cases of COVID-19.

Methods: Of the 15,376 patients who underwent head MRI/A examination called “Brain Dock” between June 2020 and June 2021, 746 patients who received a COVID-19 antibody test were evaluated. Positive and negative patients were comparatively examined for head MRI/A findings such as cerebral white matter lesions, ischemic changes, cerebral microbleeds, cerebral aneurysms, arterial stenosis, sinusitis, and other abnormal findings.

Results: Overall, 31 (4.2%) patients were COVID-19 positive, and all of them had mild infections not requiring hospitalization. There was no significant difference in patient characteristics and head MRI/A findings between positive and negative patients. All positive patients showed no particular abnormalities in the nasal findings such as olfactory bulb atrophy or thickening of the olfactory mucosa.

Conclusion: Intracranial lesions in mild patients do not show a clear difference from those in negative patients. This indicates that findings seen in common MRI/A sequences of severe patients are not likely in mild patients, supporting that there is relatively no damage to the central nervous system in mild patients.

## Introduction

Severe acute respiratory syndrome coronavirus 2 (SARS-CoV-2) is an animal-derived virus that is classified as a β-coronavirus. The infection caused by SARS-CoV-2 is called coronavirus disease 2019 (COVID-19) [1,2]. Patients mainly experience respiratory symptoms such as fever, cough, sore throat, headache, and malaise. However, neuropsychiatric symptoms such as impaired senses of smell and taste, impaired consciousness, headache, delirium, and dizziness are also observed [3]. However, complications of the central nervous system and after-effects associated with COVID-19 are yet to be completely elucidated.

Studies have been conducted to estimate the occurrence of after-effects from imaging findings in multiple areas [4]. The direct invasion of SARS-CoV-2 into the central nervous system has also been reported [5]. Autopsy studies have found ribonucleic acid transcripts in brain tissue, and viral proteins have also been identified in vascular endothelial cells of the olfactory bulb [6,7]. It is thought that, because angiotensin-converting enzyme 2 (ACE2) is most strongly expressed in nasal epithelial cells within the respiratory system, SARS-CoV-2 invades the nervous system by retracting axons in the nasal cavity, olfactory nerve, olfactory bulb, and brain stem. This results in impairments in the senses of smell and taste and infection of the medulla oblongata and pons, causing respiratory failure [8-10]. Invasion of SARS-CoV-2 into the central nervous system may cause long-term complications with poor prognosis [11]. Long-term follow-up has revealed that many people continue to experience “brain fog” and headaches after COVID-19 infection [12].

There have been multiple reports of organic abnormalities in the central nervous system that have been identified using head magnetic resonance imaging and angiography (MRI/A) and electroencephalography (EEG) in severe cases of COVID-19 requiring mechanical ventilation and intensive care management [13-15]. Virginie et al. found neuropsychiatric symptoms in one of four severely infected individuals; 71.9% had abnormal findings such as cerebral white matter lesions, multiple microhemorrhages, and localized cerebral blood flow decline on head MRI/A after infection [14]. On the other hand, most cases of COVID-19 infection are mild and do not require hospitalization, and these cases rarely require head MRI/A. As such, there are few reports of head imaging and intracranial changes in patients with mild COVID-19 infection. This study aimed to investigate whether mild COVID-19 cases are also associated with abnormal MRI/A findings that are more frequently seen in severe cases.

## Materials and methods

Study design and participants

This retrospective study was approved by the Institutional Review Board and Ethics Committee of Yokohama City University (approval no. B210300027) and was performed in accordance with the ethical standards laid down in the 1964 Declaration of Helsinki and its later amendments. The requirement for written informed consent was waived due to the retrospective study design, per the Personal Information Protection Law and National Research Ethics Guideline in Japan.

Patients who underwent a brain screening system using head MRI/A called “Brain Dock” (brain screening, SmartScan, Inc.) [16] at Medical Check Studio Tokyo Ginza and Shinjuku Clinic between June 2020 and June 2021 were included. Patients with insufficient data and in whom the COVID-19 antibody test was performed after the head MRI/A were excluded. The latest data were used for patients in whom multiple head MR images were taken. The patients were divided into antibody-tested and untested groups, and head MRI/A findings were compared between the positive and negative groups. In addition, to examine biases due to the motivation for taking antibody testing, propensity score matching was performed for the antibody untested group and the negative antibody test result group. After adjusting to the same number, a comparative analysis was performed in the same manner for the difference in head MRI/A findings depending on whether or not an antibody test was performed.

We recorded the age, sex, average body mass index (BMI), medical history (hypertension, dyslipidemia, diabetes mellitus, stroke, arrhythmia), smoking history, and drinking habits of each patient. In the positive group, the date of diagnosis of COVID-19, method of diagnosis, presence or absence of hospitalization, symptoms, symptoms remaining after infection, vaccination status, and date of vaccination were recorded. COVID-19, which did not require oxygen administration or hospitalization, was defined as “mild type.”

Antibody testing and imaging protocols

Antibody tests were performed using The Elecsys® Anti-SARS-CoV-2 S immunoassay (Roche Diagnostics International Ltd, Rotkreuz, Switzerland). The COVID-19 antibody test Elecsys® uses the electrochemiluminescence immunoassay (ECLIA) method to specifically detect SARS-CoV-2 N-IgG antibodies against nucleocapsid protein produced after being infected with COVID-19 [17,18]. At the manufacturer’s recommended cut-off index (COI) of <1.0 for defining negative infection, the detection sensitivity and specificity are 99.5% and 99.8%, respectively [17-19]. In our study, we also defined positive infection as COI≧1.0. Although there is no cross-reactivity with vaccination, our study included patients up to June 2021 before the start of large-scale vaccination in Japan.

Imaging studies were performed using a 1.5 Tesla MRI system (Vantage Elan Fast Edition; Canon Medical Systems, Tochigi, Japan). The “Brain Dock” neuroimaging protocol included the following common MRI/A sequences: T1-weighted image, T2-weighted image, fluid-attenuated inversion recovery (FLAIR) sequences, T2* weighted image, diffusion-weighted image (DWI), and MRA. Head MRI/A findings were classified into cerebral white matter lesions, ischemic changes, cerebral microbleeds, enlarged perivascular cavities, cerebral aneurysms, arterial dissections, intracranial stenoses, sinusitis, and other abnormal findings, and comparisons were made between each group. Cerebral white matter lesions consisted of deep and subcortical white matter hyperintensity and periventricular hyperintensity, with Grade 1 or higher defined as significant findings in the Fazekas classification [20]. The number of enlarged perivascular cavities, reportedly an extension of the subarachnoid space surrounding the penetrating arteries, were counted in a single axial slice in basal ganglia and centrum semiovale, according to STRIVE criteria [21]. All images were interpreted by both a radiologist and a neurosurgeon.

Statistical analysis

Categorical variables were compared using the chi-squared test or Fisher’s exact test, while continuous variables were compared using the t-test or Mann-Whitney U test, as appropriate. Missing variables were treated as deficit data, not affecting other variables. Multivariate logistic regression analyses for the positive change, defined as grade ≥1 in cerebral white matter lesions, and other abnormalities were performed in the positive and negative groups. Odds ratios (ORs) were calculated for each group. For multivariable logistic regression analysis, independent variables were selected based on existing literature, and no variable selection method such as stepwise selection was applied.

To reduce the effects of potential confounding factors in this study, a propensity score analysis was applied for the no antibody test and the negative groups. Multivariable logistic regression in which the age, sex, average BMI, medical history (hypertension, dyslipidemia, diabetes mellitus, stroke, arrhythmia), smoking history, and drinking habits of the patient were taken as independent variables were performed. After estimating the propensity score by the logistic regression, we performed 1:1 optimal nearest-neighbor matching. All statistical analyses were performed using scipy.stats (version 1.7.1) and statsmodels.api (version 0.12.2) in Python packages (version 3.9.7). P-values < 0.05 were considered to indicate statistical significance.

## Results

**Figure 1:**
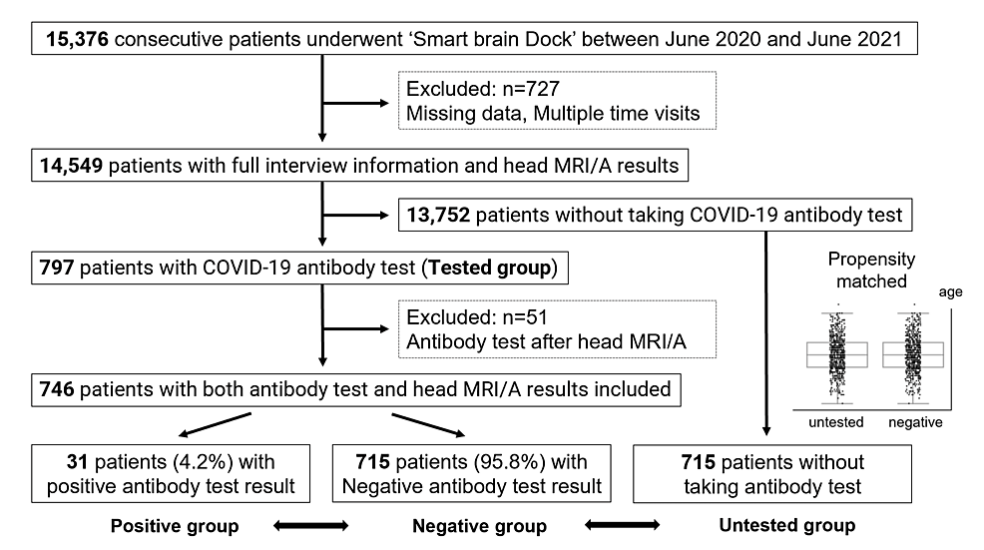
Flowchart of patient inclusion. MRI/A = magnetic resonance imaging and angiography, COVID-19 = coronavirus disease 2019

Of the 15,376 patients who underwent head MRI/A at our clinic between June 2020 and June 2021, 14,549 patients were included (Figure 1). Among the included patients, 797 patients underwent COVID-19 antibody testing. After excluding patients who underwent antibody testing after MRI/A, 746 patients remained. Of the 746 patients, 31 (4.2%) had positive antibody test results, and four were treated at hotels without oxygen. All positive patients had mild disease and did not require hospitalization. The positive group included 23 men, and the average patient age was 44.5 years (range, 20-60 years). In total, 16 patients (51.6%) were diagnosed with the infection at the time of onset using nucleic acid amplification tests (n=12) and antigen tests (n=4). The median duration from the date of a definitive diagnosis of COVID-19 to the antibody test was 91.0 days (range, 12-424 days). No patients were vaccinated before the antibody test. For patients with a definitive diagnosis, the average time from the date of definitive diagnosis to head MRI/A was 108.9 days (range, 26-260 days). Symptoms included fever in 12 patients (38.7%), malaise in five patients (16.1%), impairments in taste and smell in five patients (16.1%), headache in three patients (9.6%), and dizziness in two patients (6.5%). Meanwhile, 15 patients (48.4%) were unaware that they had the infection and only found out that they were infected after receiving the antibody test results; those patients were classified as asymptomatic. After being infected, two patients (6.5%) had residual symptoms before the head MRI/A was conducted.

Table 1 shows the results of the univariate analysis of patient information. The average age was slightly lower in the positive group (44.8±9.18 vs. 49.2±10.12; p = 0.018), but there were no significant differences between the positive and negative groups with respect to sex, BMI, pre-existing conditions (hypertension, diabetes mellitus, dyslipidemia, stroke, arrhythmia), smoking history, and drinking history. Table 2 shows the results of the multivariate analysis of MRI/A findings. There were no significant differences between the two groups: the presence or absence of cerebral white matter lesions (Grade 1 or higher; p=0.218), ischemic changes (p=0.522), cerebral microbleeds (p=0.767), enlarged perivascular cavities (p=0.891), cerebral aneurysm (p=0.893), arterial dissection, intracranial stenosis (p=0.998), sinusitis (p=0.302), and other abnormal findings (p=0.142).

**Table 1:** Patient characteristics * Untested group was propensity-matched with the negative antibody test group. BMI = body mass index

Characteristic	Tested group		P-value (A vs. B)	Untested group	P-value (B vs. C)
	Positive	Negative		*	
	(A; n=31)	(B; n=715)		(C; n=715)	
Age (y)	44.8 (20-60)	49.2 (21-94)	0.018	49.2 (21-94)	0.998
Sex			0.651		1
Male	23 (74.7%)	492 (68.8%)		492 (68.8%)	
Female	8 (25.3%)	223 (31.2%)		223 (31.2%)	
median BMI	23.0 (16.8-27.3)	23.9 (15.9-42.0)	0.147	23.5 (16.0-37.9)	0.041
Past history					
Hypertension	7 (22.6%)	200 (28.0%)	0.652	201 (28.1%)	1
Diabetes mellitus	0 (0%)	38 (5.3%)	0.368	35 (4.9%)	0.81
Dyslipidemia	3 (9.7%)	150 (21.0%)	0.194	146 (20.4%)	0.845
Stroke	0 (0%)	5 (0.7%)	0.511	2 (0.3%)	0.449
Arrhythmia	2 (6.5%)	25 (3.5%)	0.71	23 (3.2%)	0.883
Smoking history	13 (41.9%)	297 (41.5%)	0.887	316 (44.2%)	0.336
Drinking history	26 (83.9%)	525 (73.4%)	0.277	542 (75.8%)	0.331

**Table 2:** Patient MRI findings * Untested group was propensity-matched with negative antibody test group. MRI = magnetic resonance imaging, DSWMH = Deep and Subcortical White Matter Hyperintensity, PVH = Periventricular Hyperintensity

MRI Finding	Tested group		P-value (A vs. B)	Untested group	P-value (B vs. C)
	Positive	Negative		*	
	(A; n=31)	(B; n=715)		(C; n=715)	
Cerebral white matter lesions (Grade 1 or higher)	10 (32.3%)	323 (45.2%)	0.218	316 (44.2%)	0.75
DSWMH (Grade 1 or higher)	10 (32.3%)	301 (42.1%)	0.367	296 (41.4%)	0.83
PVH (Grade 1 or higher)	1(3.2%)	102 (14.3%)	0.139	90 (12.6%)	0.394
Cerebral ischemic change	0 (0%)	28 (3.9%)	0.522	21 (2.9%)	0.383
Cerebral microbleeds	1 (3.2%)	17 (2.4%)	0.767	10 (1.4%)	0.243
Enlarged perivascular cavities (Grade 1 or higher)	9 (29.0%)	228 (31.9%)	0.891	249 (34.8%)	0.262
Cerebral aneurysm	0 (0%)	10 (1.4%)	0.893	8 (1.1%)	0.813
Arterial dissection	0 (0%)	0 (0%)		0 (0%)	
Intracranial stenosis	1 (3.2%)	11 (1.5%)	0.998	15 (2.1%)	0.554
Sinusitis	6 (19.4%)	212 (29.7%)	0.302	203 (28.4%)	0.641
Other abnormalities	6 (19.4%)	241 (33.7%)	0.142	235 (32.9%)	0.779

Nasal findings such as olfactory bulb atrophy or thickening of the olfactory mucosa were confirmed retrospectively in all COVID-19-positive cases, and there were no particular abnormalities. Of the 31 patients with positive antibody tests, four patients had head MRI/A taken before and after being infected. In all these patients, no changes including the nasal findings were observed in the MRI/A before and after being infected.

**Figure 2:**
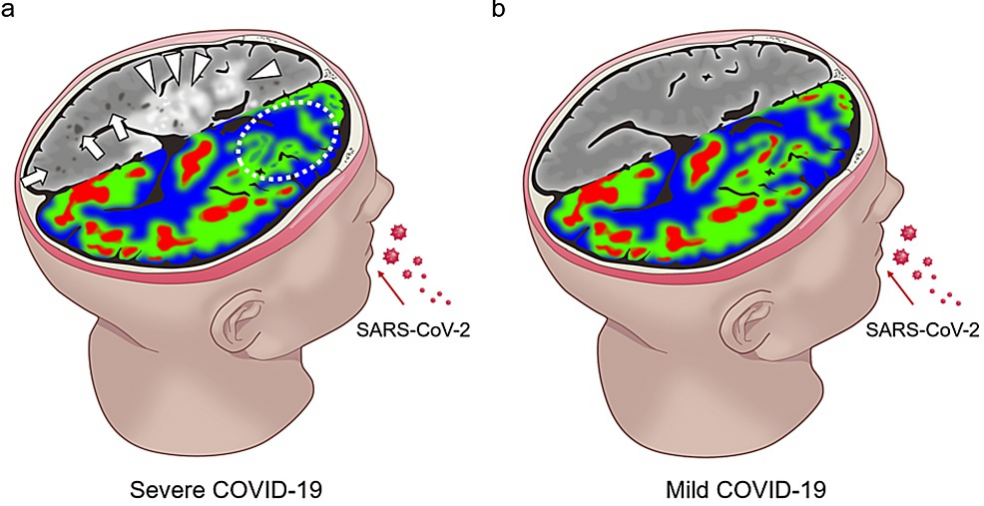
Illustrative summary of frequent findings on head magnetic resonance imaging (MRI). This is our original illustration. (a) Head MRI illustration in severe cases showing high-grade cerebral white matter lesions (arrowhead), multiple cerebral microbleeds (arrow), and hypoperfusion in the frontal lobe (circle). (b) Head MRI illustration in mild cases showing no particular abnormalities in the brain.

Furthermore, 715 patients who were propensity-matched with 715 patients with negative antibody test results were extracted from among 13,752 patients who had not taken an antibody test. Only the mean BMI was slightly lower in the unexamined group (23.5±3.39 vs. 23.9±3.62; p = 0.041), but the other items were adjusted to almost the same tendency (Table 1). Multivariate analysis (Table 2) showed no significant difference in the presence or absence of findings such as cerebral white matter lesions (grade ≥1, p=0.750), ischemic changes (p=0.383), cerebral microbleeds (p=0.243), enlarged perivascular cavities (p=0.262), cerebral aneurysm (p=0.813), arterial dissection, intracranial stenosis (p=0.554), sinusitis (p=0.641), and other abnormalities (p=0.779). Figure 2a is a summary of the commonly reported head MRI findings of severely ill patients [13,15]. Our study revealed that there are no clear brain MRI findings in patients with mild COVID-19 infection (Figure 2b).

## Discussion

The information on the mechanism by which COVID-19 causes neurological complications and neurological symptoms is updated daily, but there are still many unclear points about the possibility of after-effects on the central nervous system. Although several randomized clinical trials have investigated symptoms, imaging findings, and treatment methods in moderate or severe COVID-19 cases, there are still few reports on mild cases, which account for the majority of infected individuals.

Similar to severe acute respiratory syndrome and middle east respiratory syndrome, COVID-19 has been reported to, directly and indirectly, affect the nervous system, with patients exhibiting a variety of neuropsychiatric symptoms [4,22]. The mechanisms by which SARS-CoV-2 invades the central nervous system may include: 1) direct invasion of SARS-CoV-2 from the nasal cavity into the central nervous system via the olfactory bulb; 2) infected monocytes pass through the blood-brain barrier and infects the glial cells and neurons; 3) SARS-CoV-2 binds to the ACE2 receptor on the endothelial cells of the blood-brain barrier and invades the central nervous system; and 4) SARS-CoV-2 invades the central nervous system via the peripheral nervous system [8,9]. Furthermore, hypoxemia due to respiratory failure, encephalopathy due to an abnormal immune response, and cerebral infarction due to a thrombotic tendency are also mechanisms by which SARS-CoV-2 affects the brain. One study found SARS-CoV-2 in the brains of 58 out of 87 patients in autopsy [6], providing additional evidence that SARS-CoV-2 directly infiltrates the brain.

According to a report summarizing the non-specific neurological symptoms of COVID-19 inpatients headache, dizziness, anorexia, fatigue, and impaired smell and taste were reported in 8%-42%, 12%, 40%, 11%-44%, and 5%, respectively [23]. Regarding central neurological disorders, there are many reports of complications of a stroke. SARS-CoV-2 is thought to cause a stroke by damaging the vascular endothelium, exacerbating inflammation and thrombotic tendency. Reports on patients requiring intensive care in China, Europe, and the United States have stated that 5.9-76.8% had stroke complications, and approximately 80% had cerebral infarction [24-26]. Additionally, autopsy reports of 10 patients showed microbleeds and infarction in the blood vessels of the cerebral parenchyma, despite patients not presenting specific neurological symptoms [7].

There are multiple reports of abnormal findings on common head MRI/A sequences in COVID-19 patients who required hospitalization [13-15]. In a cross-sectional study of head MRI/A findings of 125 COVID-19 in patients with neuropsychiatric symptoms across the UK, 77 (62%) had a cerebrovascular disorder, 57 (74%) had cerebral infarction, nine (12%) had a cerebral hemorrhage, and one (1%) patient had central nervous system vasculitis [27]. A total of 39 patients (31%) had psychiatric symptoms, of whom nine patients (23%) had nonspecific encephalopathy and seven patients (18%) had encephalitis. In a French multi-center retrospective observational study of head MRI findings which included 190 patients who required hospitalization for at least oxygen administration, abnormalities were found in 37 patients (19.5%) [13]. The main MRI findings were increased signals in unilateral FLAIR and DWI in 16 (43%), multiple cerebral white matter lesions in 11 (30%), and cerebral microbleeds in nine (24%) patients. Chougar et al. reported that among 73 COVID-19 patients who had neuropsychiatric symptoms during hospitalization and head MRI/A images taken, 58.9% had abnormal findings two to four weeks after onset, 47.7% had abnormalities of cerebral perfusion, 23.3% had cerebral infarction, 11.3% had cerebral microbleeds, and 5.5% had multiple cerebral white matter lesions [15]. Thus, despite several reports on head MRI/A findings of hospitalized COVID-19 patients, there are few reports of head MRI/A for mildly ill, non-hospitalized patients-who account for the majority of infected individuals because head MRI/A is rarely performed in patients who do not require hospitalization and have few neuropsychiatric symptoms.

To investigate the future risk of after-effects, it is important to examine the presence or absence of brain parenchymal abnormalities after infection even in mild cases [11]. In Japan, “Brain Dock,” in which both head MRI and MRA are conducted, is performed as a head screening for healthy individuals. We used “Brain Dock” to evaluate MR findings after mild COVID-19 infection in this study.　The number of our patients who tested positive was comparable to the positivity rate in Japan at that time. All patients had a mild infection and were not hospitalized, and almost half were asymptomatic. The average duration from the date of diagnosis to the antibody test and head MRI/A were 113.4 and 108.9 days, respectively, indicating that the evaluation was performed after some time had passed since being infected.

A comparison of imaging findings showed no significant difference in findings (e.g., cerebral white matter lesions, cerebral microbleeds, and cerebral infarction, which are major findings in severe cases) between individuals who tested positive and negative. No patient in the positive group showed abnormal nasal findings such as olfactory bulb atrophy or thickening of the olfactory mucosa, which are easily damaged by COVID-19 infection. This suggests that there is relatively no damage to the central nervous system in mild than severe cases. Among the patients in the positive group, we were able to monitor the images of four patients before and after being infected. All patients had fever at the time of infection, and symptoms such as headache (two patients) and impairments in the senses of taste and smell (three patients) were observed. No infection-associated changes in brain parenchymal findings were observed.

Furthermore, to evaluate biases regarding the motivation for taking an antibody test, we examined whether there was a difference in imaging findings between those who had a negative antibody test result and who had only head MRI/A without undergoing an antibody test. The results showed that there was no significant difference in the imaging findings. Hence, the motivation for taking an antibody test did not significantly affect the results of our study. Considering the above findings, there may be a few specific changes in common head MRI/A sequences after infection with COVID-19 in mild cases.

Reportedly, there are relatively few complications of neuropsychiatric symptoms in mild cases [28,29]. Liu et al. found that the average amount of virus in COVID-19 mild cases is approximately 60 times lower than in severe cases, and virus clearance is observed at an earlier stage; thus, the risk of direct infiltration into the central nervous system is relatively small [29]. Our results support previous reports based on imaging findings. Notably, Douaud et al. reported brain volume reductions of 0.2%-2.0% even in mild cases of COVID-19, particularly in the gray matter of the orbitofrontal cortex and parahippocampal gyrus, suggesting that even mild cases may cause neurological damage, although these are very minimal and cannot be detected by common MRI/A sequences [30]. However, the cause of sequelae - such as brain fog and olfactory abnormalities - may be very subtle brain atrophy that cannot be detected by common head MRI/A sequences, concurring with our results. Based on the above, if neurological symptoms remain after mild COVID-19 infection, long-term follow-up-including brain volume measurement-is recommended even if there are no abnormalities in common head MRI/A sequences.

The limitations of our study include the use of antibodies instead of nuclear acid amplification tests to detect infections. The advantage of using an antibody test is that asymptomatic cases, which account for nearly half of the mild cases, can be examined. However, the accuracy of information such as the elapsed time from infection-is lacking. Additionally, due to the nature of the Brain Dock-which is for the imaging of healthy individuals-contrast-enhanced MRI, blood flow and brain volume evaluation could not be performed in this study. Further, it is possible that abnormal findings could have occurred during the acute phase of infection but were not observed on head MRI/A as imaging was performed sometime after infection. However, it is essential to evaluate the irreversible findings in considering the possibility of after-effects. Thus, the analysis of abnormal findings on common head MRI/A sequences sometime after infection, as in our study, is meaningful.

## Conclusions

In conclusion, our study has shown that organic abnormalities that are seen on common MRI/A sequences in severe cases do not appear in mild cases, indicating relatively no damage to the central nervous system in mild patients. However, given that microreductions in brain volume have been reported even in mild cases, further studies regarding the aftereffects of COVID-19 on the nervous system are needed. Additional investigation and follow-up of symptoms will be necessary in the future.

## References

[1] Bi Q, Wu Y, Mei S (2020). Epidemiology and transmission of COVID-19 in 391 cases and 1286 of their close contacts in Shenzhen, China: a retrospective cohort study. Lancet Infect Dis.

[2] Li Q, Guan X, Wu P (2020). Early transmission dynamics in Wuhan, China, of novel coronavirus-infected pneumonia. N Engl J Med.

[3] Hensley MK, Markantone D, Prescott HC (2022). Neurologic manifestations and complications of COVID-19. Annu Rev Med.

[4] Bungenberg J, Humkamp K, Hohenfeld C (2022). Long COVID-19: objectifying most self-reported neurological symptoms. Ann Clin Transl Neurol.

[5] Desforges M, Le Coupanec A, Dubeau P, Bourgouin A, Lajoie L, Dubé M, Talbot PJ (2019). Human coronaviruses and other respiratory viruses: underestimated opportunistic pathogens of the central nervous system?. Viruses.

[6] Puelles VG, Lütgehetmann M, Lindenmeyer MT (2020). Multiorgan and renal tropism of SARS-CoV-2. N Engl J Med.

[7] Fabbri VP, Foschini MP, Lazzarotto T (2021). Brain ischemic injury in COVID-19-infected patients: a series of 10 post-mortem cases. Brain Pathol.

[8] Sungnak W, Huang N, Bécavin C (2020). SARS-CoV-2 entry factors are highly expressed in nasal epithelial cells together with innate immune genes. Nat Med.

[9] De Santis G (2020). SARS-CoV-2: a new virus but a familiar inflammation brain pattern. Brain Behav Immun.

[10] Yoshida M, Worlock KB, Huang N (2022). Local and systemic responses to SARS-CoV-2 infection in children and adults. Nature.

[11] Xie Y, Xu E, Bowe B, Al-Aly Z (2022). Long-term cardiovascular outcomes of COVID-19. Nat Med.

[12] Tabacof L, Tosto-Mancuso J, Wood J (2022). Post-acute COVID-19 syndrome negatively impacts physical function, cognitive function, health-related quality of life, and participation. Am J Phys Med Rehabil.

[13] Kremer S, Lersy F, de Sèze J (2020). Brain MRI findings in severe COVID- 19: a retrospective observational study. Radiology.

[14] Lambrecq V, Hanin A, Munoz-Musat E (2021). Association of clinical, biological, and brain magnetic resonance imaging findings with electroencephalographic findings for patients with COVID-19. JAMA Netw Open.

[15] Chougar L, Shor N, Weiss N (2020). Retrospective observational study of brain MRI findings in patients with acute SARS-CoV-2 infection and neurologic manifestations. Radiology.

[16] Yamasaki T, Ikawa F, Hidaka T (2021). Prevalence and risk factors for brain white matter changes in young and middle-aged participants with Brain Dock (brain screening): a registry database study and literature review. Aging (Albany NY).

[17] Riester E, Findeisen P, Hegel JK (2021). Performance evaluation of the Roche Elecsys Anti-SARS-CoV-2 S immunoassay. J Virol Methods.

[18] Higgins V, Fabros A, Kulasingam V (2021). Quantitative measurement of anti-SARS-CoV-2 antibodies: analytical and clinical evaluation. J Clin Microbiol.

[19] Sethuraman N, Jeremiah SS, Ryo A (2020). Interpreting diagnostic tests for SARS-CoV-2. JAMA.

[20] Fazekas F, Chawluk JB, Alavi A, Hurtig HI, Zimmerman RA (1987). MR signal abnormalities at 1.5 T in Alzheimer's dementia and normal aging. AJR Am J Roentgenol.

[21] Wardlaw JM, Smith EE, Biessels GJ (2013). Neuroimaging standards for research into small vessel disease and its contribution to ageing and neurodegeneration. Lancet Neurol.

[22] Li YC, Bai WZ, Hashikawa T (2020). The neuroinvasive potential of SARS-CoV2 may play a role in the respiratory failure of COVID-19 patients. J Med Virol.

[23] Gupta A, Madhavan MV, Sehgal K (2020). Extrapulmonary manifestations of COVID-19. Nat Med.

[24] Xiong W, Mu J, Guo J (2020). New onset neurologic events in people with COVID-19 in 3 regions in China. Neurology.

[25] Lodigiani C, Iapichino G, Carenzo L (2020). Venous and arterial thromboembolic complications in COVID-19 patients admitted to an academic hospital in Milan, Italy. Thromb Res.

[26] Oxley TJ, Mocco J, Majidi S (2020). Large-vessel stroke as a presenting feature of covid-19 in the young. N Engl J Med.

[27] Varatharaj A, Thomas N, Ellul MA (2020). Neurological and neuropsychiatric complications of COVID-19 in 153 patients: a UK-wide surveillance study. Lancet Psychiatry.

[28] Brodin P (2021). Immune determinants of COVID-19 disease presentation and severity. Nat Med.

[29] Liu Y, Yan LM, Wan L (2020). Viral dynamics in mild and severe cases of COVID-19. Lancet Infect Dis.

[30] Douaud G, Lee S, Alfaro-Almagro F (2022). SARS-CoV-2 is associated with changes in brain structure in UK Biobank. Nature.

